# Unlocking the Hepatoprotective Potential of *Cyperus rotundus* Through Edible Vinegar Processing: A Study on Functional Ingredient Enhancement

**DOI:** 10.1002/fsn3.71850

**Published:** 2026-05-04

**Authors:** Gao Jia‐He, Lin Li‐Ting, Liu Yue‐Han, Wang Fu‐Chao, Liu Jun‐Tong, Gao Tian‐Hui

**Affiliations:** ^1^ School of Pharmacy Qilu Medical University Zibo Shandong China; ^2^ School of Pharmacy Chengdu University of Traditional Chinese Medicine; Key Laboratory of Standardization of Chinese Medicine (Chengdu University of Traditional Chinese Medicine), Ministry of Education; Lab for Innovation & Effective Uses of Chinese Drug Germplasm Resources Chengdu Sichuan China

**Keywords:** α‐cyperone, *Cyperus rotundus L.*, gut‐liver axis, TLR4/NF‐κB pathway, vinegar‐processed

## Abstract

*Cyperus rotundus* L. (CR), a globally pervasive weed with dual agricultural and medicinal significance, undergoes vinegar‐processed (VCR) to enhance its hepatoprotective properties. This study investigated the phytochemical and functional changes induced by vinegar processing and elucidated the role of α‐cyperone, a key bioactive sesquiterpene, in modulating gut‐liver axis homeostasis. UPLC‐Q‐Exactive Orbitrap MS and HPLC analyses revealed a significant increase in α‐cyperone content post‐vinegar processing. In thioacetamide‐induced acute and chronic liver injury models, VCR significantly outperformed CR in reducing serum ALT and AST, hepatic TNF‐α and IL‐6, and oxidative stress, while restoring gut barrier integrity via up‐regulation of ZO‐1 and occludin. Molecular docking supported α‐cyperone's high‐affinity binding to the TLR4/NF‐κB pathway, corroborated by western blot and immunofluorescence. Gut microbiota analysis demonstrated VCR's capacity to reverse dysbiosis, notably enriching Bacteroidetes and improving microbial diversity. These findings highlight edible vinegar processing as a sustainable strategy to transform agricultural waste into a functional food‐grade intervention for liver health, mediated by α‐cyperone‐driven modulation of the gut‐liver axis.

## Graphical abstract

*Cyperus rotundus* L. (CR) and vinegar‐processed CR (VCR) were compared in mouse models of thioacetamide (TAA)‐induced acute and chronic liver injury. VCR exhibited better hepatoprotective effects than raw CR. Composition analysis revealed 35 components in VCR, among which α‐cyperone was present at higher levels than in raw CR. Molecular docking predicted favorable binding affinity between α‐cyperone and key proteins of the TLR4/NF‐κB pathway. These predictions were further validated by immunofluorescence and Western blotting, confirming that α‐cyperone regulates the TLR4/NF‐κB pathway. Given that this pathway is a critical axis of the gut‐liver axis, the protective effects of α‐cyperone on intestinal barriers were also evaluated. The results showed that α‐cyperone protects both the mechanical and biological barriers of the intestine. Collectively, CR and VCR alleviate TAA‐induced liver injury through α‐cyperone‐mediated suppression of TLR4/NF‐κB signaling and preservation of gut barrier integrity.

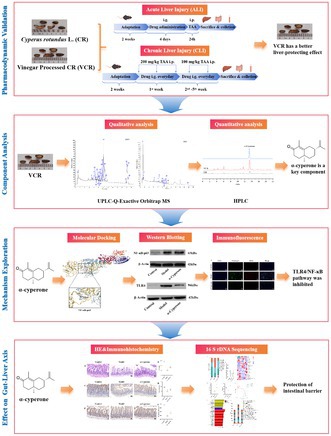

## 1 Introduction

Vinegar is a common fermented product (Fernandez‐Perez et al. 2010), has long been used as a condiment and in traditional and modern medicine worldwide (Xia, Zhang, Duan, et al. 2020). Documented medicinal applications of vinegar span millennia: Hippocrates (460–377 bce) recommended it for ulcerations and sores (Johnston and Gaas 2006). In 8th‐century Japan, Samurai warriors ingested it as a fortifying tonic (Budak et al. 2014). Sung Tse, founder of Chinese forensic medicine in the 10th century, utilized vinegar‐sulfur mixtures for infection prevention. By the 18th century, U.S. practitioners treated conditions including poison ivy, croup, stomach ache, and high fever with vinegar (Ho et al. 2016). Recently, research showed that vinegar extract could be used as a novel gut microbiota manipulator against alcohol‐induced liver damage (Xia, Tong, Xia, et al. 2020; Xia, Zhang, Duan, et al. 2020; Xia, Zhang, Li, et al. 2020). There was also a study reported that sodium acetate which is abundantly present in vinegar could suppress hepatocellular carcinoma progression (Hou 2023).

China is the earliest country in the world to produce vinegar from cereal (Li et al. 2021) such as corn, sorghum, millet, and rice. The medicinal value of vinegar is further demonstrated by its essential role as an excipient in the processing of traditional Chinese medicine (TCM). TCM processing is listed among China's first batch of National Intangible Cultural Heritage items and represents one of the most distinctive traditional pharmaceutical technologies with independent intellectual property rights in China (Jia 2019). When TCMs are processed with vinegar at specific ratios through heating methods such as soaking, stir‐frying, steaming, or boiling, their therapeutic effects are modified. For instance, vinegar‐processed Cyperi Rhizoma (CR), a representative vinegar‐processed herbal medicine, whose qi‐regulating and pain‐relieving effects are strengthened with concurrent improvement in hepatocyte membrane permeability for its active constituents (Li and Hu 2012).

CR is the dried rhizome of *Cyperus rotundus* L., a perennial herbaceous plant belonging to the Cyperaceae family. Characterized by high photosynthetic efficiency, this species exhibits genetic diversity, strong tolerance to adverse climatic conditions, high reproductive capacity, ease of dispersal, and competitive superiority, including allelopathic effects (Lati et al. 2011). These adaptations enable it to thrive across diverse agroclimatic zones, making it widely distributed yet difficult to control selectively (Durigan 2005). Recognized as one of the “World's Ten Worst Weeds”, CR exerts dual detrimental effects on crops through both aboveground foliage and subterranean rhizome systems, infesting over 40 food and cash crops‐including corn, sugarcane, soybeans, and fruit trees‐with documented global yield reductions of 20% ~ 90% in agriculture and horticulture (Salgado et al. 2002). Paradoxically, this aggressive weed is also a renowned medicinal herb, first documented in the *Ming Yi Bie Lu* during the Liang Dynasty (1869 ad). TCM attributes to it the functions of regulating menstruation, alleviating pain, moving qi, and resolving depression, earning it the epithets “Commander of Qi Disorders” and “Foremost Herb in Women's Medicine”. Vinegar‐processed CR, first recorded in *Yeshi Luyanfang* in the Song Dynasty (1186 ad), has been clinically proven in TCM to potentiate its therapeutic efficacy in liver qi regulation and pain relief. Furthermore, the medicinal use of CR for gastrointestinal ailments has been documented since antiquity across regions such as South Korea, Japan, Egyptand (Lu et al. 2022) and Thailand. Modern research has further demonstrated that CR exhibits hepatoprotective effects when combined with other Chinese herbal medicines, showing potential in preventing and treating liver diseases such as hepatic fibrosis, fatty liver, and cirrhosis (Liang et al. 2022). CR possesses a complex phytochemical profile, with terpenoids and flavonoids being the most prominent. Terpenoids, particularly sesquiterpenes, dominate the volatile oil fraction, which constitutes approximately 0.65%–1.4% of the rhizome (Janaki et al. 2018). Key sesquiterpenes identified include α‐cyperone, cyperenone, cyperotundone, and caryophyllene oxide. Flavonoids such as luteolin, quercetin, and kaempferol have also been isolated, along with various alkaloids, sterols, and other minor components. Among these, α‐cyperone has emerged as a signature and quantitatively significant constituent of CR. It is consistently reported as one of the most abundant compounds in the essential oil, often comprising 22%–25% of the total volatile fraction, and is considered a chemotaxonomic marker for the species (Wang et al. 2022). Beyond its abundance, α‐cyperone demonstrates a wide array of pharmacological activities. Notably, it has been alleviated Crohn's disease‐like colitis in mice via TLR4/NF‐κB pathway modulation (Zhang, Zhang, Song, et al. 2024), while in Parkinson's disease models it exerts neuroprotection by activating Nrf2/HO‐1 and suppressing NF‐κB signaling‐mediated neuroinflammation (Huang et al. 2023).

Liver injury is a common pathological condition caused by various factors, including hepatitis viruses, ethanol, drug/toxin exposure, cholestasis, lipid or heavy metal deposition, and immune dysregulation. It can be classified as acute or chronic based on disease progression and, if left untreated, may advance to liver failure, fibrosis, or even hepatocellular carcinoma. Globally, liver disease is a leading global cause of death, responsible for over 2 million fatalities annually, with cirrhosis and liver cancer disproportionately affecting young adults and costing the U.S. healthcare system $32.5 billion in 2016 alone (Devarbhavi et al. 2023). Importantly, early‐stage liver injury is often reversible, making preventive and therapeutic research critically valuable. Emerging evidence suggested that modulation of the gut‐liver axis represented a promising therapeutic strategy for hepatoprotection (Albillos et al. 2020). Our preliminary studies using multiple animal models demonstrated that vinegar‐processing of TCMs such as *Curcuma phaeocaulis* Val. and *Curcuma Longa* L. significantly enhances their hepatoprotective effects (Gan et al. 2018; Gao et al. 2022; Gao, Lin, et al. 2024; Gao, Wang, et al. 2024) by regulate gut‐liver axis including TLR4/MyD88/NF‐κB pathway. In this study, we will investigate the phytochemical differences and hepatoprotective efficacy of CR before and after vinegar processing, further elucidate the therapeutic potential of rice vinegar as a pharmaceutical excipient and provide experimental evidence for clinical liver injury treatment.

## 2 Materials and Methods

### 2.1 Materials and Reagents

CR was supplied by Kangmei Pharmaceutical Co. Ltd. and verified by the professor of TCM identification in Qilu Medical University. Thioacetamide (TAA, no. H37020766, 98%) was formed by Beijing Solarbio Technology Co. Ltd. (Beijing, China). Silybin meglumine Tablets (SM, no. 20220029) was obtained from Hunan Qianjin Xieli Pharmaceutical Co. Ltd. (Hunan, China). Bifendate pills (BF, no. 19J231239) was obtained from Wanbangde Pharmaceutical Group Co. Ltd. (Zhejiang, China). α‐Cyperone (no. YRX051‐231001) was acquired from Chengdu Yirui Biotechnology Co. Ltd. (Sichaun, China). Sodium carboxymethylcellulose (CMC‐Na, no. 130110006100) was purchased from Shandong Usolf Chemical Technology Co. Ltd. (Shandong, China). Standards of ethanol, methanol, formic acid and phosphoric acid were HPLC grade and provided by Sigma‐Aldrich. Elisa kits (IL‐1β, IL‐6, TNF‐α, IL‐17A) were purchased from Thermo Fisher Scientific (China) Co. Ltd. (Shanghai, China), Biochemical kits (ALT, AST, ALB) were obtained from Rayto Life Sciences Co. Ltd. (Shenzhen, China), SOD and MDA kits were purchased from Nanjing Jiancheng Technology Co. Ltd. (Nanjing, China), TBiL kit and bovine serum albumin (BSA) were acquired from Wuhan Servicebio Biotechnology Co. Ltd. (Wuhan, China). Antibodies for NF‐κB‐p65, TLR4, β‐Actin were obtained from HuaAn Biotechnology Co. Ltd. (Hangzhou, China), while the Occludin and ZO‐1 antibody was obtained from Wuhan SANYING Biotechnology (Wuhan. China).

### 2.2 Preparation of CR and Vinegar‐Processed CR Samples

Vinegar‐processed CR (VCR) was prepared according to the previously optimized processing protocol (Lu et al. 2012): 100 g CR was mixed thoroughly with 25 mL rice vinegar diluted in 15 mL purified water, then moistened for 6 h. The mixture was dry‐fried at 150°C for 8 min to yield the final VCR. The CR and VCR samples were prepared according to the research group's previously established method (Sheng et al. 2016): 100 g coarse powder of CR and VCR was macerated in 600 mL of 95% ethanol for 12 h, followed by 20‐min ultrasonication and filtration. The residue was re‐extracted with another 600 mL of 95% ethanol (20‐min ultrasonication, filtration). The combined filtrates were concentrated at 45°C until alcohol‐free, followed by lyophilization for subsequent use. HPLC was used to determine the quality control of CR and VCR samples. It was found that CR samples contain 0.26 ± 0.009 mg/g α‐cyperone, VCR samples contain 1.04 ± 0.0003 mg/g α‐cyperone.

### 2.3 Animal Experiment

C57BL/6 mice were purchased from Jinan Pengyue Experimental Animal Breeding Co. Ltd. (Shandong, China) with free access to adequate food and water under standardized environmental conditions at 28.8°C ± 3°C and humidity of 17.6% ± 5%. Animal experiments were performed under the approval of the Institutional Animal Care and Use Committee of Qilu Medical University (permit YXLL2025D065).

#### 2.3.1 Acute Liver Injury (ALI) in Mice

After 2‐week acclimatization, 56 mice were randomly assigned to 7 groups (*n* = 8) using a random number generator. (i) The control group was given 10 mL/kg of 0.9% normal saline by i.g. every day for 4 days. (ii) The model group was given 10 mL/kg of 0.9% normal saline by i.g. every day for 4 days. (iii) 5 g/kg of CR sample was given to mice by i.g. every day (CR‐H) for 4 days. (iv) 2.5 g/kg of CR sample was given to mice by i.g. every day (CR‐L) for 4 days. (v) 5 g/kg of VCR sample was given to mice by i.g. every day (VCR‐H) for 4 days. (vi) 2.5 g/kg of VCR sample was given to mice by i.g. every day (VCR‐L) for 4 days. (vii) 60 mg/kg of SM was given to mice by i.g. every day (SM) for 4 days. After pretreatment for 1 h on the day of the acute liver injury experiment, each mouse except for the normal mice was given 200 mg/kg of TAA by i.p. The control group was given the same dose of 0.9% normal saline by i.p. Based on established rat dosing regimen (Sheng et al. 2016), the doses were first scaled to equivalent mouse doses and then evaluated in a series of pre‐experiments. We found that when the doses were 2.5–5 g/kg, the degree of liver lesions was alleviated without significant adverse effects. Finally, in this experiment, similar doses of CR were used to determine the dosage of 2.5 and 5 g/kg as the low and high groups.

#### 2.3.2 Chronic Liver Injury (CLI) in Mice

After 2‐week acclimatization, 56 mice were randomly assigned to 7 groups (*n* = 8) using a random number generator. Except the control group, other groups received 100 mg/kg TAA three times per week by i.p. during the first week, and received 200 mg/kg TAA twice per week by i.p. from the second to fifth week. (i) The control group was given 10 mL/kg of 0.9% normal saline by i.g. every day for weeks 2–5. (ii) The model group was given 10 mL/kg of 0.9% normal saline by i.g. every day for weeks 2–5. (iii) The CR‐H group was given 5 g/kg of CR sample by i.g. every day for Weeks 2–5. (iv) The CR‐L group was given 2.5 g/kg of CR sample by i.g. every day for Weeks 2–5. (v) The VCR‐H group was given 5 g/kg of VCR sample by i.g. every day for Weeks 2–5. (vi) The VCR‐L group was given 2.5 g/kg of VCR sample by i.g. every day for Weeks 2–5. (vii) 75 mg/kg of BF was given to mice by i.g. every day (BF) for Weeks 2–5.

### 2.4 UPLC‐Q‐Exactive Orbitrap MS Analysis of VCR

A 10 mL of methanol was used to dissolve 0.5 g VCR samples under ultrasonic extraction for 30 min separately. Then, the supernatant was passed through a 0.22 μm filter membrane, and 2 μL sample of the filtered solution was injected into the LC–MS for analysis. VCR samples partitioning occurred upon an Ultimate UHPLC XB‐C18 column (2.1 × 150 mm, 1.8 μm) (Welchmat, Shanghai, China), with 0.1% formic acid in water (A) and acetonitrile (B) as the mobile phase at a flow rate of 0.3 mL/min. The elution scheme was delineated thusly: from 0 to 2 min, 8% to 15% B; from 2 to 15 min, 15% to 35% B; from 15 to 18 min, 35 to 60% B; from 18 to 21 min, 60% to 90% B; from 21 to 23 min, 90% B; from 23 to 25 min, 90% to 8% B. All samples were analyzed in positive and negative ion modes. Nitrogen was used as the auxiliary and sheath gas, with flow rates of 10 L/min and 40 L/min, respectively. In positive mode, the ion spray had a voltage of 3.8 kV, while in negative mode, it had a voltage of 3.0 kV. The scanning mass ratio remained within the mass spectrum of *m/z* 80–1200. The LC–MS data analysis was conducted using Compound Discoverer analytical software (Thermo Fisher Scientific). The experimental data were cross‐referenced with online chemical databases (Mz Cloud and Mz Vault) and relevant literature to identify potential components.

### 2.5 HPLC Analysis of VCR

A 10 mL of methanol was used to dissolve 0.5 g VCR samples under ultrasonic extraction for 30 min separately. Then, the supernatant was passed through a 0.22 μm filter membrane, and 10 μL sample of the filtered solution was injected into the HPLC‐DAD for analysis. VCR samples partitioning occurred upon a HPLC C18‐PAQ column (4.6 × 250 mm, 5 μm) (COSMOSIL, Kyoto, Japan), with water (A) and methanol (B) as the mobile phase at a flow rate of 1.0 mL/min. The detection wavelength was 243 nm. α‐Cyperone was dissolved in methanol as standard and used to establish the linear regression equation and concentration‐peak area correlation.

### 2.6 Molecular Docking Verification

The crystal structure of α‐cyperone is obtained from Protein Data Bank. AutoDock 4.2.6 (The Scripps Research Institute, La Jolla, CA, USA) was employed for virtual molecular docking. The docked models were visually analyzed using PyMOL 2.4.1 (DeLano Scientific LLC, San Carlos, CA, USA).

### 2.7 Observation of Liver Phenotype

Fresh intact livers from all mice were collected and weighed to calculate the liver index. The liver size, surface color, texture, tactile sensation, hardness, and the presence of fine granular formations within the tissue were recorded.

### 2.8 H&E Staining, Immunofluorescence and Immunohistochemistry

Mouse liver and ileum tissue was fixed in 4% paraformaldehyde for 24 h, dehydrated, soaked in xylene, embedded in paraffin, sectioned with a paraffin slicer, stained with H&E, mounted, and observed and captured using Panoramic Scan 150 (3DHISTECH Ltd., Hungary) and CaseViewer (3DHISTECH Ltd., Hungary). Two experimenters, blinded to the treatment group assignments, evaluated the coded slides using the Ishak scoring system (Ishak et al. 1995). For each slide, five random fields were observed under a light microscope to assess the degree of liver injury and inflammatory activity. The structural morphology of length of intestinal villi, depth of crypt, and the villus height‐to‐crypt depth ratio (V/C ratio) were assessed using Image‐Pro Plus 6.0 software. Immunofluorescence and immunohistochemistry analysis involved incubation of the sections in 3% BSA and overnight exposure to primary antibodies at 4°C. Subsequently, the liver and ileum sections were washed with PBS and treated with secondary antibodies. Imaging was captured using an inverted fluorescence microscope (Nikon, Japan). Immunostaining demonstrated positive outcomes. The ImageJ software was employed to assess the rate of expression positivity.

### 2.9 Determination of ALT, AST, ALB, TBiL, SOD, and MDA

The contents of ALT, AST, ALB, TBiL, SOD, and MDA in serum and liver were determined by commercial test kits.

### 2.10 Elisa Analysis of Inflammatory Factor

The levels of IL‐1β, IL‐6 and TNF‐α in the liver tissue were measured following the instructions provided in the Elisa kit manual.

### 2.11 Fecal Microbiota Analysis by 16 S rDNA Sequencing

Genomic DNA (gDNA) was extracted and purified from fecal samples using a commercial DNA extraction kit following the manufacturer's protocol. The integrity of gDNA was verified by 0.8% agarose gel electrophoresis, and its concentration was quantified using a spectrophotometer. The V4 hypervariable region of the bacterial 16S rRNA gene was amplified using indexed primers 515F (5′‐GTGYCAGCMGCCGCGGTAA‐3′) and 806R (5′‐GGACTACHVGGGTWTCTAAT‐3′). The PCR reaction mixture (50 μL total volume) comprised 5 μL 10× PCR Buffer for KOD‐Plus‐Neo, 5 μL 2 mmol/L dNTPs, 3 μL 25 mmol/L MgSO₄, 1.5 μL each of forward (U515F) and reverse (U806R) primers (10 μmol/L), 1 μL KOD‐Plus‐Neo polymerase (1 U/μL), 2 μL template DNA, and 31 μL nuclease‐free H₂O. Amplicons were sequenced on an Illumina MiSeq platform (PE 250), and the raw data were processed using QIIME2 (version 2020.2) for bioinformatic analyses, including differential abundant species identification, α‐ and β‐diversity metrics, and linear discriminant analysis effect size to evaluate microbial community structural and functional variations.

### 2.12 Western Blotting Analysis

Proteins from the liver tissue were extracted. The total protein concentration was detected using the BCA assay kit. Following SDSPAGE, PVDF transferring, proteins were blocked with 5% skim milk to prevent nonspecific binding. Afterwards, the membrane was incubated overnight at 4°C with the primary antibodies (anti‐TLR4, anti‐NF‐κB‐p65, and anti‐β‐Actin) at a 1:1000 dilution, followed by incubation with secondary antibodies at room temperature for 1 h.

### 2.13 Statistical Analysis

Statistical analyses were performed using IBM SPSS Statistics 29.0 (SPSS Inc., Chicago, IL, USA) and GraphPad Prism 9.0 (GraphPad Software, San Diego, CA, USA). Continuous variables are presented as mean ± standard deviation (SD). The normality of data distribution was verified using the Shapiro–Wilk test. The homogeneity of variances was assessed using the Brown‐Forsythe test. Subsequently, one‐way ANOVA was applied for group comparisons, followed by Fisher's Least Significant Difference (LSD) test. A confidence level of 95% was adopted, and statistical significance was defined as *p* < 0.05.

## 3 Results

### 3.1 Therapeutic Effect of CR and VCR on ALI Mice

**FIGURE 1:**
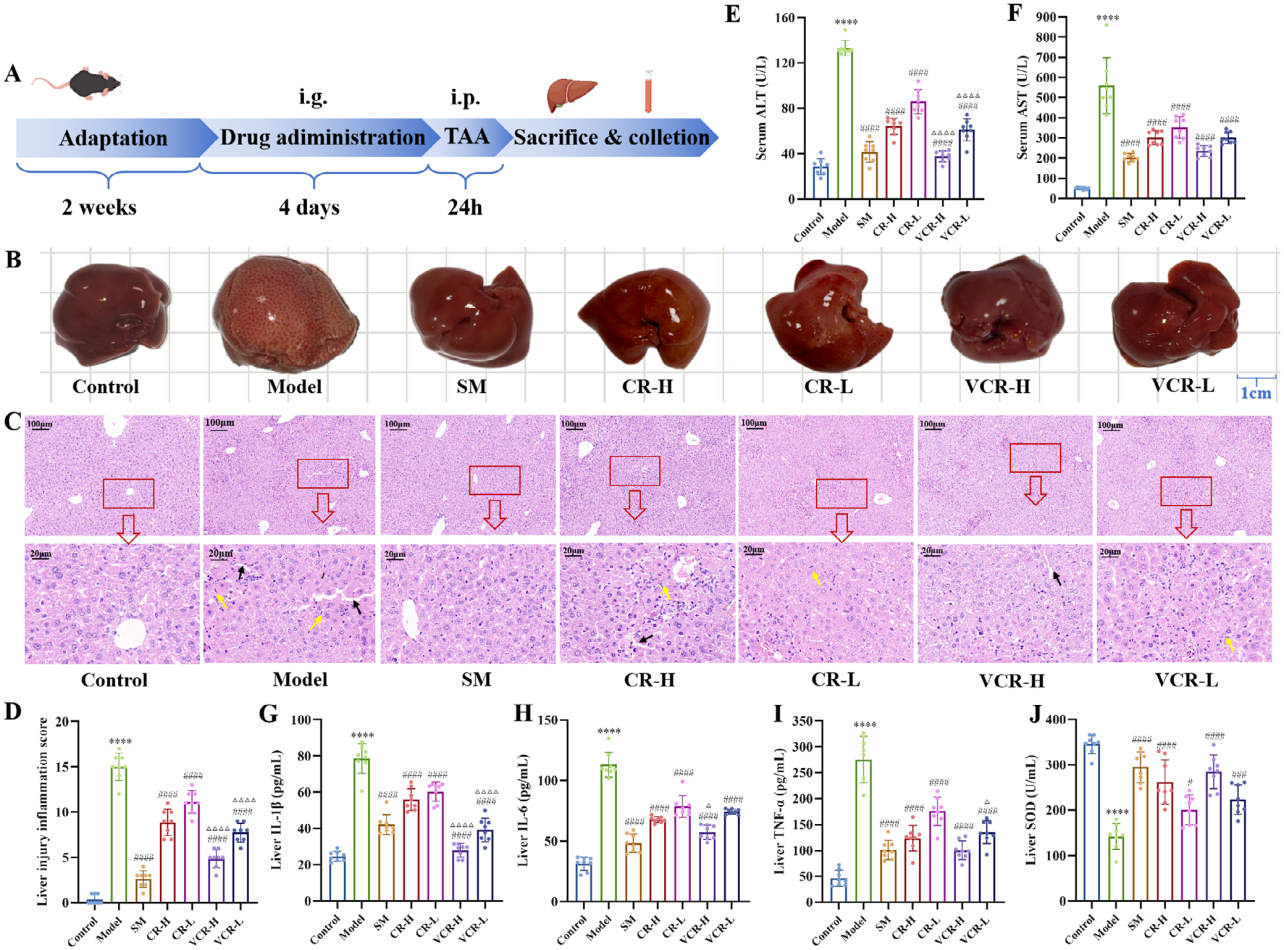
Therapeutic effect of CR and VCR on ALI mice. (A) Schematic of the experimental design. (B) Photograph of the livers. (C–D) H&E staining of liver tissue with 100× and 400× magnification. (E, F) Serum levels of ALT and AST. (G–J) Liver levels of IL‐1β, IL‐6, TNF‐α and SOD. *p < 0.05, **p < 0.01, ***p < 0.001, ****p < 0.0001 versus control; ^#^ *p* < 0.05, ^##^ *p* < 0.01, ^###^ *p* < 0.001, ^####^ *p* < 0.0001 versus model; ^△^ *p* < 0.05, ^△△^ *p* < 0.01, ^△△△^ *p* < 0.001, ^△△△△^ *p* < 0.0001 vs equal‐dose CR and VCR; ns: Not significant; same as below.

After molding and treating as described in Figure 1A, both CR and VCR demonstrated hepatoprotective effects, with VCR exhibiting superior efficacy against liver injury. As shown in Figure 1B, the liver volume in the model group was slightly enlarged compared to the control group, with a pale color and a rough surface covered with dark red granular spots. However, these pathological morphological changes were significantly improved in all treatment groups. Figure 1C revealed that in the model group, hepatic architecture was disrupted, characterized by dilated sinusoidal spaces (black arrows) and extensive inflammatory cell infiltration (yellow arrows). CR and VCR treatment significantly reduced the area of liver inflammation compared with the model group. Meanwhile, CR and VCR treatment significantly reduced the levels of ALT and AST in serum, and the levels of IL‐1β, IL‐6, and TNF‐α in liver. Furthermore, VCR demonstrated a more pronounced amelioration of inflammatory damage in liver tissue (Figure 1D–I). The VCR‐H group reduced IL‐6 levels in liver tissue more significantly than the CR‐H group (*p* = 0.0494), while the VCR‐L group reduced TNF‐α levels in liver tissue more significantly than the CR‐L group (*p* = 0.0480). CR and VCR treatment significantly elevated hepatic SOD level (Figure 1J), VCR also demonstrated a more favorable antioxidant profile compared to CR treatments.

### 3.2 Therapeutic Effect of CR and VCR on CLI Mice

**FIGURE 2:**
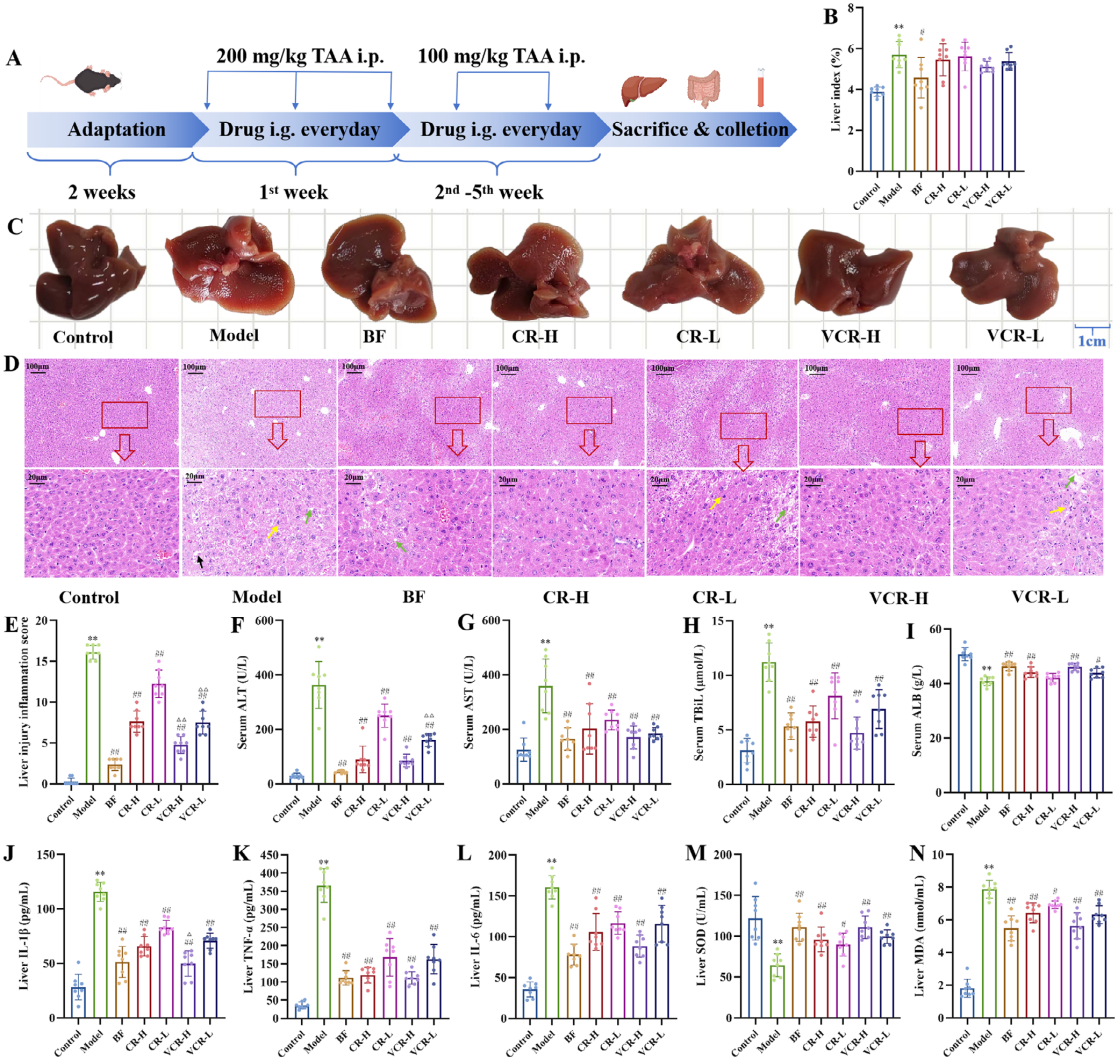
Therapeutic effect of CR and VCR on CLI mice. (A) Schematic of the experimental design. (B) The relative liver index (liver/body weight). (C) Photograph of the livers. (D, E) H&E staining of liver tissue with 100× and 400× magnification. (F–I) Serum levels of ALT, AST, TBiL, and ALB. (J–N) Liver levels of IL‐1β, IL‐6, TNF‐α, SOD, and MDA.

CLI mice model was established to evaluate the hepatoprotective effects of CR and VCR (Figure 2A). Compared to the model group, the BF group could significantly reduce liver index (*p* = 0.0127), while both CR and VCR treatment groups showed no significant changes in liver index (Figure 2B). In terms of macroscopic liver appearance, the CR and VCR treatment groups exhibited darker liver color and reduced orange‐peel‐like lesions on the surface (Figure 2C). H&E staining further revealed that all CR and VCR treatment groups significantly alleviated vacuolar hepatocyte necrosis (green arrows), reduced hepatic plate fissures (black arrows), and decreased inflammatory infiltration (yellow arrows). Notably, VCR demonstrated superior efficacy compared to CR. The liver inflammation score of the VCR‐H group was significantly lower than that of the CR‐H group (*p* = 0.0001), while the VCR‐L group was also significantly lower than that of the CR‐H group (*p* < 0.0001) (Figure 2D,E). Additionally, both CR and VCR treatment significantly reduced serum levels of ALT, AST, and TBIL while increasing ALB (Figure 2F–I), decreased liver tissue levels of IL‐1β, TNF‐α, IL‐6, and MDA, while increasing SOD activity (Figure 2J–N). Further analysis revealed that the VCR‐L group had significantly lower serum ALT than the CR‐L group (*p* = 0.0024), while the VCR‐H group had significantly increased hepatic IL‐1β levels than the CR‐H group (*p* = 0.0397), indicating VCR enhanced anti‐inflammatory effects against CLI.

### 3.3 Chemical Constituents of VCR

Animal experiments demonstrated enhanced hepatoprotective effects of VCR. To further analyze its active pharmaceutical ingredients, UPLC‐Q‐Exactive Orbitrap MS was used to qualitatively analyze the ingredients in VCR. 35 compounds were identified (Table 1), with α‐cyperone exhibiting a marked increase in relative abundance post‐vinegar processing. Since α‐cyperone served as a key quality marker and was specified as a quantitative standard in the Hong Kong Chinese Materia Medica Standards, α‐cyperone was subsequently subjected to HPLC quantification. The analysis revealed substantially higher levels in VCR (1.04 ± 0.0003 mg/g) compared to CR (0.26 ± 0.009 mg/g) (*p* < 0.0001) (Figure S1).

**TABLE 1:** Mass spectrometry information of the main compounds in VCR.

NO	*t*_R_ (min)	Formula	Name	Measured	Predicted	Error (ppm)	Model	MS^2^
1	1.26	C_6_H_6_O_3_	5‐HMF	126.03169	126.03199	2.37	[M + H]^+1^	53.03935, 68.01372, 80.05001, 108.04471
2	1.291	C_6_H_12_O_6_	Hex‐2‐ulose	180.06339	180.0631	−1.59	[M‐H]^−1^	56.01387, 68.05017, 95.06088, 110.07162, 120.05592, 137.08229, 161.08228, 179.06352
3	1.34	C_7_H_12_O_6_	1,3,4,5‐Tetrahydroxycyclohexanecarboxylic acid	192.06339	192.06312	−1.38	[M‐H]^−1^	61.26457, 68.11764, 76.99754, 90.62870, 98.26428, 114.16311, 135.95348, 203.67303
4	6.256	C_9_H_8_O_3_	p‐Coumaric acid	164.04734	164.04689	−2.78	[M‐H]^−1^	52.27831, 61.98708, 65.03851, 93.03354, 119.04932, 128.25308, 136.06833, 162.36836
5	7.44	C_15_H_22_O	α‐Cyperone	218.16707	218.16715	0.37	[M + H]^+1^	65.03927, 79.05476, 91.05472, 105.07030, 115.05453, 131.08572, 149.09625, 159.11681, 177.12758, 219.13817
6	7.47	C_15_H_20_O_3_	Amiloxate	248.14124	248.14157	1.31	[M + H]^+1^	67.05484, 79.05492, 91.05465, 105.07034, 119.08576, 128.06207, 145.10098, 161.09639, 189.12758, 231.13788
7	8.03	C_9_H_16_O_4_	Azelaic acid	188.10486	188.10437	−2.6	[M‐H‐H_2_O]^−1^	60.88486, 78.08066, 90.58708, 110.37042, 136.30025, 164.79376, 173.70125
8	8.76	C_10_H_18_O_2_	5‐Decanolide	170.13068	170.13019	−2.88	[M‐H]^−1^	57.00285, 61.51239, 84.15775, 97.32698, 114.45109
9	8.86	C_15_H_24_O	Butylated hydroxytoluene	220.18272	220.18271	−0.04	[M + H‐H_2_O]^+1^	55.05503, 67.05492, 81.07046, 91.05479, 133.10129, 147.11685, 161.13274, 203.17960
10	8.941	C_10_H_10_O_4_	Ferulic acid	194.05791	194.05812	1.1	[M + H]^+1^	89.50731, 100.07616, 113.96403, 122.09676, 141.95880, 154.99034, 158.96149, 182.98541, 217.15895
11	9.06	C_6_H_14_N_4_O_2_	DL‐Arginine	174.11168	174.11182	0.81	[M + H]^+1^	53.03927, 65.03918, 91.05476, 105.07034, 115.05454, 131.08583, 144.05696, 159.08058
12	9.75	C_15_H_20_O_2_	(+)‐Alantolactone	232.14633	232.14651	0.78	[M + H‐H_2_O]^+1^	53.03933, 67.05486, 81.07041, 91.05477, 105.07018, 115.05451, 128.06215, 145.10123, 159.11690
13	9.81	C_15_H_18_O_3_	(−)‐α‐Santonin	246.12559	246.12588	1.15	[M + H]^+1^	65.03912, 85.02866, 91.05469, 115.05458, 128.06215, 143.08582, 153.06985, 169.10117, 183.11678, 211.11179
14	10.13	C_18_H_34_O_5_	(−)‐Pinellic acid	330.24062	330.24168	3.2	[M‐H]^−1^	70.06577, 88.07611, 106.08669, 114.38479, 312.32736
15	10.24	C_20_H_39_NO_2_	Oleoylethanolamide	325.29808	325.29819	0.35	[M + H]^+1^	83.86623, 103.08998, 142.01564, 161.58804, 202.39024, 327.15659, 340.60429
16	10.27	C_15_H_22_O	Cyperenone	218.16707	218.16725	0.85	[M + H]^+1^	67.05489, 79.05482, 91.05472, 105.07021, 119.08582, 131.08571, 163.07541, 173.13297, 219.17316
17	10.3	C_12_H_14_O_2_	Butylphthalide	190.09938	190.09957	0.98	[M + H]^+1^	55.05493, 65.03915, 79.05470, 91.05464, 105.07013, 115.05418, 128.06224, 135.08119, 191.14345
18	10.787	C_15_H_22_O	(+)‐Nootkatone	218.16707	218.16727	0.95	[M + H]^+1^	55.05498, 67.05494, 79.05478, 91.05477, 105.07020, 145.10147, 159.11690, 201.16341, 219.17346
19	10.85	C_15_H_18_O_2_	Dehydrocostus lactone	230.13068	230.13018	−2.17	[2 M + Na]^+1^	67.05486, 79.05476, 91.05468, 105.07017, 119.08575, 133.10117, 175.14801, 203.14296, 231.13805
20	10.9	C_7_H_6_O_4_	Protocatechuic acid	154.02661	154.02601	−3.86	[M‐H]^−1^	56.97704, 76.96892, 84.12482, 94.90913, 110.79220, 136.16196, 154.94707, 164.91660
21	10.925	C_18_H_34_O_4_	Dibutyl sebacate	314.24571	314.24681	3.5	[M‐H]^−1^	60.81033, 73.48490, 90.63680, 114.15697, 164.64500, 232.29721, 303.58273, 337.73419
22	11.42	C_15_H_22_O_2_	Valerenic acid	234.16198	234.16214	0.69	[M + H]^+1^	67.05492, 79.05479, 91.05479, 105.07022, 119.08578, 133.10135, 161.09607, 189.16393, 235.16904
23	11.46	C_9_H_18_O_2_	1‐Nonanoic acid	158.13068	158.12993	−4.76	[M‐H]^−1^	59.93096, 65.03926, 76.93360, 91.05483, 105.07033, 115.05453, 128.06223, 144.09344, 159.11703
24	11.54	C_15_H_24_	(E,E)‐α‐Farnesene	204.1878	204.18799	0.93	[M + H]^+1^	55.05497, 67.05483, 79.05471, 91.05471, 109.06506, 123.08067, 145.10127, 205.15855
25	11.63	C_10_H_10_O_2_	Safrole	162.06808	162.06809	0.06	[M + H]^+1^	54.93811, 65.03916, 79.05481, 91.05472, 105.07018, 133.06483, 148.08809, 163.11203
26	11.805	C_16_H_32_O_3_	Juniperic acid	272.23514	272.2361	3.5	[M‐H]^−1^	54.41642, 60.82968, 83.89676, 97.45103, 151.74672, 172.46086, 192.05341, 203.40033, 268.94217
27	12.17	C_12_H_16_O_2_	Ibufenac	192.11503	192.11527	1.23	[M + H]^+1^	55.05496, 67.05490, 79.05481, 91.05473, 107.08592, 135.11678, 149.13240, 191.14355, 203.65816
28	12.43	C_30_H_48_O_3_	Oleanolic acid	456.36035	456.36116	1.79	[M + ACN + H]^+1^	86.09697, 104.10740, 184.07346, 313.27512, 402.66464, 478.32983, 496.34137
29	12.62	C_14_H_28_O_3_	3‐Hydroxymyristic acid	244.20384	244.2045	2.67	[M‐H]^−1^	69.70176, 73.23833, 84.07782, 97.49066, 114.02345, 134.38170, 173.29120
30	12.8	C_4_H_6_O_5_	(±)‐Malic acid	134.02152	134.02198	3.38	[M + H]^+1^	53.00291, 65.03928, 79.05465, 91.05470, 105.07015, 115.05438, 124.71139, 135.08069
31	21.58	C_16_H_32_O_2_	Palmitic acid	256.24023	256.24074	1.99	[M‐H]^−1^	56.99582, 69.41790, 84.15701, 101.33929, 157.18755, 271.46399
32	21.89	C_15_H_20_O	Hexyl cinnamaldehyde	216.15142	216.15123	−0.86	[M + H]^+1^	55.28090, 67.05472, 80.94839, 91.05485, 105.07014, 119.08629, 157.10118, 189.16399, 217.15828
33	22.09	C_12_H_20_O_2_	Geranyl acetate	196.14633	196.14656	1.15	[M + H‐H_2_O]^+1^	56.01387,68.05017, 95.06088, 110.07162, 120.05592, 137.08229
34	22.14	C_10_H_16_	(±)‐Limonene	136.1252	136.12537	1.23	[M + H]^+1^	54.03460, 68.05013, 73.20700, 84.09816, 95.06083, 120.97273, 136.08704, 142.15833, 155.52914
35	22.64	C_5_H_9_NO_2_	L‐Proline	115.06333	115.06367	2.96	[M + H]^+1^	58.02948, 69.07067, 84.02731, 88.83099, 97.43393, 104.23566, 131.33014

### 3.4 Molecular Docking of α‐Cyperone and Liver Injury Core Target Protein

**FIGURE 3:**
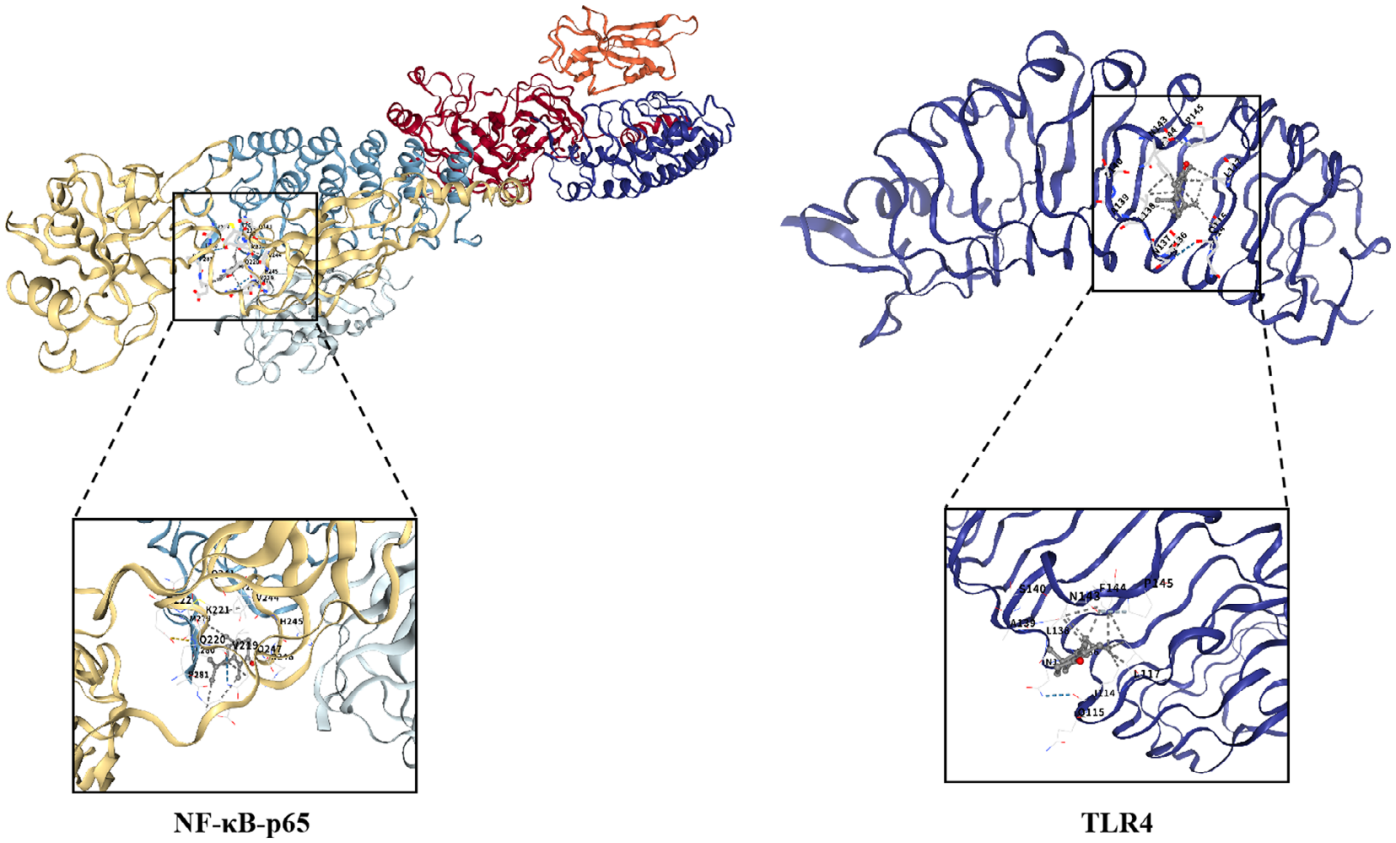
Molecular docking results of α‐cyperone with liver injury core target proteins NF‐κB‐p65 (binding energy: −6.8 kcal·mol^−1^) and TLR4 (binding energy: −6.0 kcal·mol^−1^).

Our research group has previously identified that NF‐κB‐p65 and TLR4 are core proteins of the TLR4/NF‐κB pathway, a pivotal signaling pathway in the gut‐liver axis. Consequently, molecular docking technology was employed to evaluate the interaction potential of α‐cyperone with NF‐κB‐p65 and TLR4 proteins. A binding energy < −5 kcal·mol^−1^ indicates favorable binding activity, where lower values suggest stronger ligand‐protein (Cheng et al. 2025) interaction. The results suggested that α‐cyperone exhibited a strong binding affinity to both NF‐κB‐p65 and TLR4, with binding energies of −6.8 and −6.0 kcal·mol^−1^, respectively. For NF‐κB‐p65, the docking cavity volume was 3597 Å^3^, with the grid center positioned at (−3, 79, 106) and grid box dimensions of 32 Å × 25 Å × 19 Å. For TLR4, the cavity volume was 154 Å^3^, the grid center was set at (17, −14, 8), and the grid box size was 19 Å × 19 Å × 19 Å. Molecular docking visualization of α‐cyperone with two core targets further supported these findings (Figure 3). This favorable outcome highlights the spontaneous binding capacity of α‐cyperone to these pivotal proteins, reinforcing its strong interaction potential and key role in modulating gut‐liver axis related molecular mechanisms. These predictive findings await further validation through in vivo animal studies.

### 3.5 NF‐κB‐p65 and TLR4 Modulation by α‐Cyperone via Animal Experiment

**FIGURE 4:**
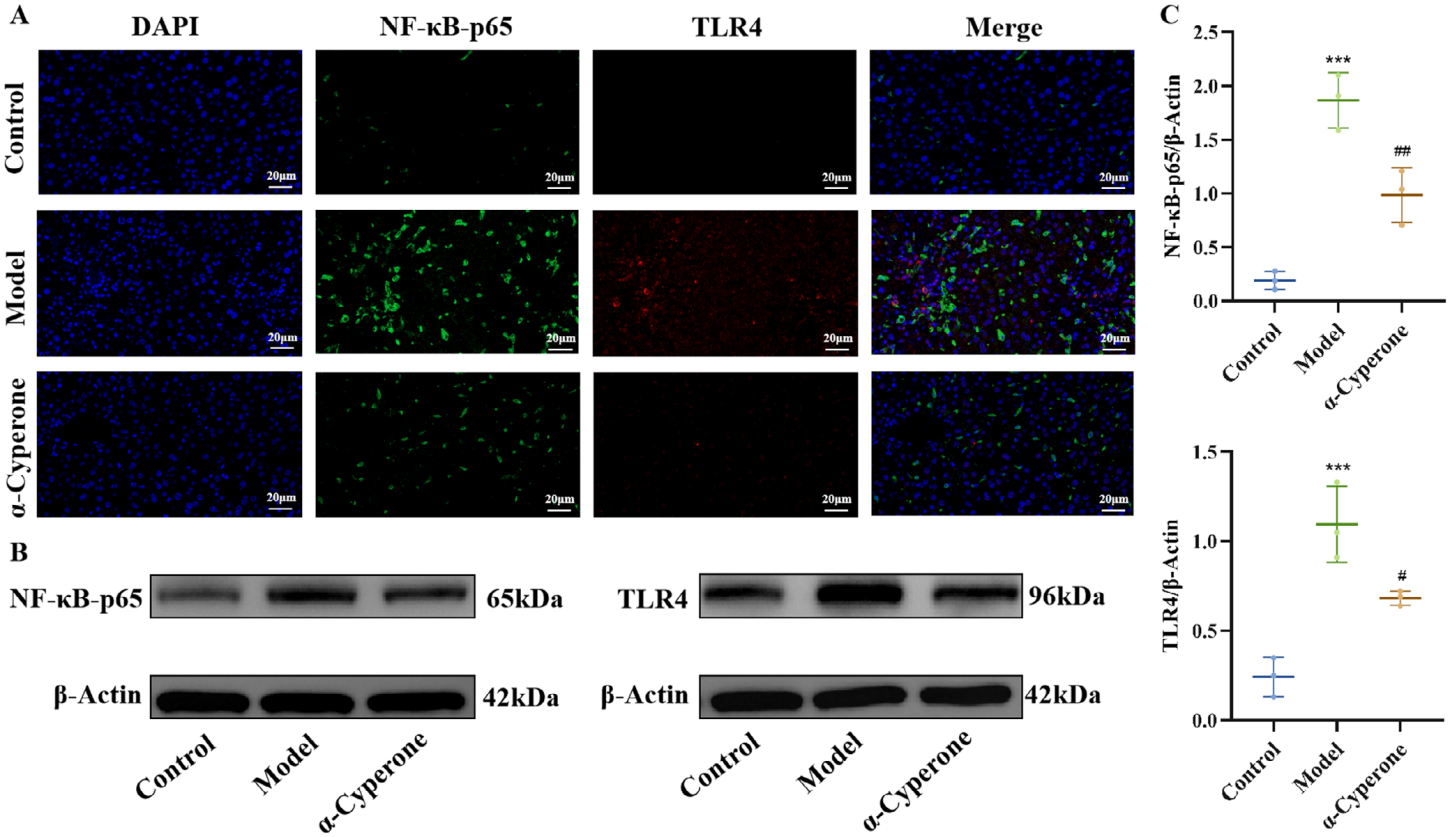
TLR4/NF‐κB pathway were involved in the mechanism by which α‐cyperone improves liver injury. (A) NF‐κB‐p65 and TLR4 expression was measured using immunofluorescence. (B, C) The protein expression levels of NF‐κB‐p65 and TLR4 were detected using western blot.

Western blot and immunofluorescence analysis demonstrated that chronic inflammatory liver injury significantly upregulated hepatic NF‐κB‐p65 and TLR4 phosphorylation compared to control groups (Figure 4A). Importantly, α‐cyperone treatment (85 mg/kg) markedly reduced both NF‐κB‐p65 (*p* = 0.0058) and TLR4 (*p* = 0.0266) expression (Figure 4B,C), supporting its role in attenuating gut‐liver axis hyperactivation and exerting hepatoprotection. According to the reported dosage of α‐cyperone in other animal models (Xia, Tong, Xia, et al. 2020; Xia, Zhang, Duan, et al. 2020; Xia, Zhang, Li, et al. 2020), we performed preliminary experiments to identify an effective and safe dose of 85 mg/kg, which was ultimately adopted in this study.

### 3.6 α‐Cyperone Ameliorates Gut Barrier Impairment

**FIGURE 5:**
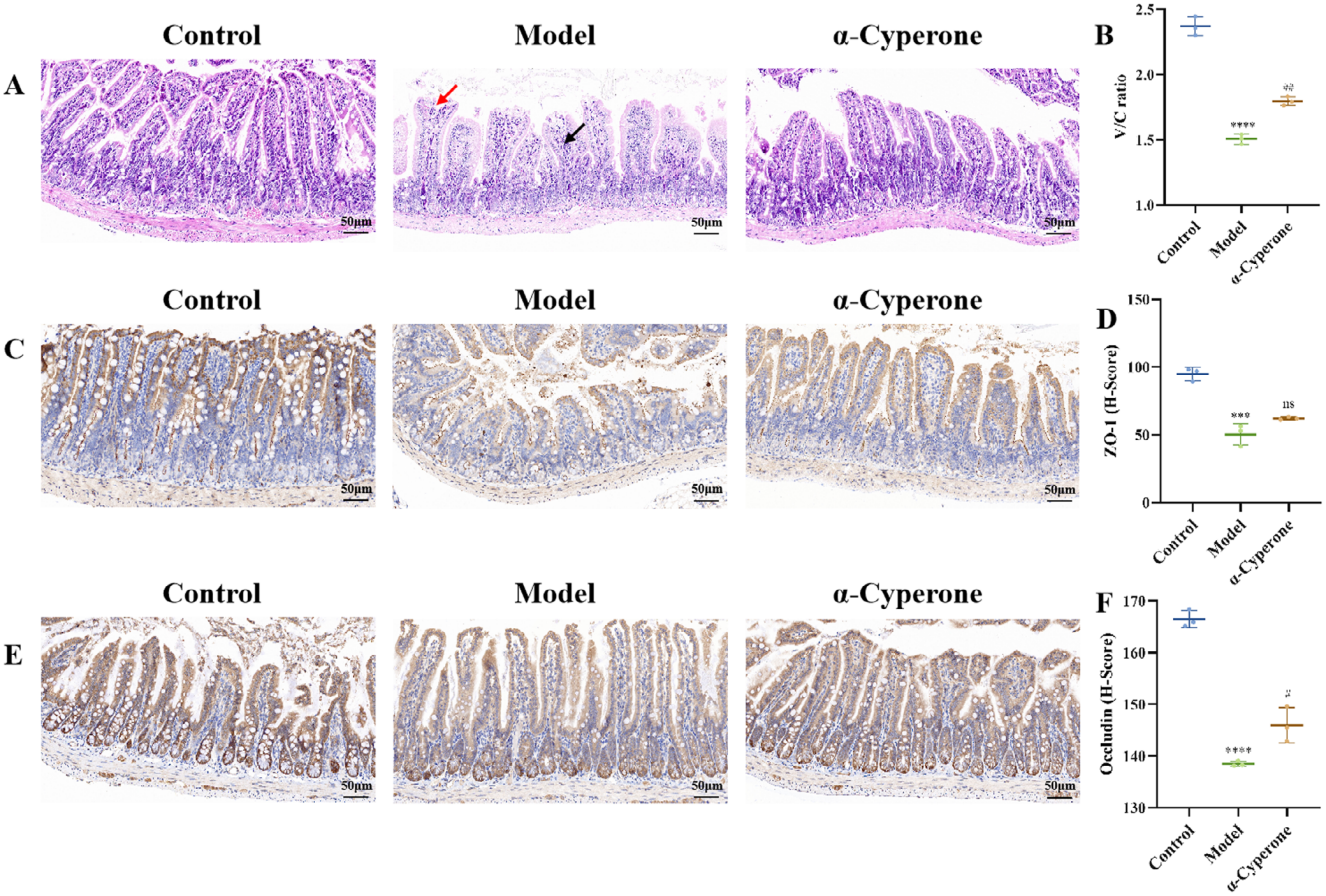
α‐Cyperone improved the intestinal barrier of liver injury mice. (A) H&E staining of ileum tissue with 200× magnification. (B) V/C ratio. (C) Positive expression of ZO‐1 protein with 200× magnification. (D) The histochemistry score (*H*‐Score) levels of ZO‐1. (E) Positive expression of occludin protein with 200× magnification. (F) The *H*‐Score levels of occludin.

The hepatoprotective effects of α‐cyperone may regulate intestinal barrier function via the gut‐liver axis. α‐Cyperone ameliorated structural damage and epithelial sloughing (red arrow) in ileum tissues of the model group, along with localized inflammatory cell infiltration (black arrow) (Figure 5A). It significantly increased the V/C ratio (*p* = 0.0011) (Figure 5B). Furthermore, α‐cyperone upregulated intestinal expression of tight junction proteins Occludin (*p* = 0.0145) and ZO‐1 (*p* = 0.0790) (Figure 5C–F), thereby attenuating liver injury.

### 3.7 α‐Cyperone Regulates Gut Microbiota

**FIGURE 6:**
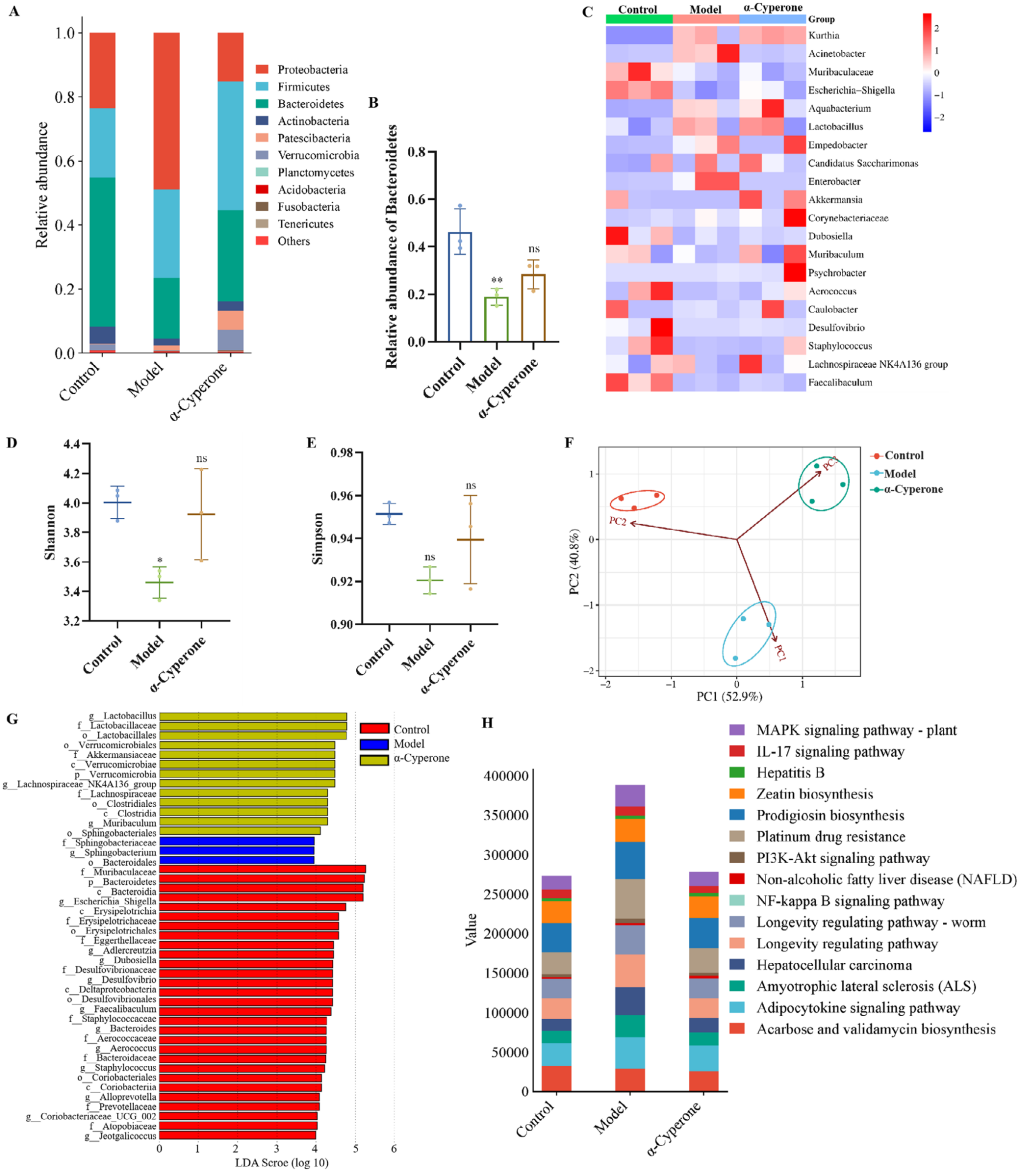
α‐Cyperone changed the composition of gut microbiota in mice. (A) Microbial distributions at the phylum level. (B) Relative abundance of *Bacteroidetes*. (C) Heat map at species level. (D) α Diversity analysis–Shannon, which is a diversity index based on information theory, incorporating both species richness and evenness, with higher values indicating greater community diversity. (E) α Diversity analysis–Simpson, which is one of the indices used to estimate microbial diversity, and the results in the figure were presented as 1‐D (where D is Simpson's Index), with higher values indicating greater diversity. (F) PCA analysis. (G) LDA score, which allows for the differential testing of taxa between two or more groups and identifies taxa that contribute significantly to intergroup differences, and longer bars indicate greater importance of the corresponding taxon in the figure. Furthermore, p, c, o, f, g stands for phylum, class, order, family, and genus, respectively. (H) Functional prediction based on KEGG pathway.

A total of 30 phyla were detected in all samples, with Bacteroidetes, Firmicutes, and Proteobacteria showing the highest abundance (Figure 6A). At the phylum level, liver injury significantly reduced the abundance of Bacteroidetes (*p* = 0.0065, Figure 6B), a phylum crucially involved in maintaining intestinal homeostasis and resisting pathogen invasion. Treatment with α‐cyperone restored Bacteroidetes abundance while enhancing microbial diversity at the species level (Figure 6C). α Diversity analysis demonstrated that the model group could reduce the Shannon (*p* = 0.0356) and Simpson indices, while α‐cyperone treatment improved both richness and diversity indices of gut microbiota in liver injury mice (Figure 6D,E). Principal component analysis (PCA) revealed distinct clustering patterns, wherein α‐cyperone treatment substantially attenuated liver injury‐induced microbial community shifts (Figure 6F). LEfSe analysis (LDA score > 2) identified 45 differentially abundant microbial taxa across the three groups (Figure 6G), indicating α‐cyperone's remodeling effects on gut microbiota composition in the liver injury model. Functional profiling using KEGG database annotation identified 377 pathways at level 3, with the top 15 most abundant pathways shown in Figure 6H. These findings suggest that α‐cyperone's hepatoprotective effects may be mediated through both structural restoration of microbial equilibrium and functional modulation of microbiome activities.

## 4 Discussion

Vinegar is not only a common condiment but also holds significant medicinal value. With its abundance of disease‐preventing phenolic compounds, vinegar extract acts as a gut microbiota modulator that enhances intestinal homeostasis and counters alcohol‐induced liver damage (Xia, Tong, Xia, et al. 2020; Xia, Zhang, Duan, et al. 2020; Xia, Zhang, Li, et al. 2020). Beyond its direct medicinal applications, vinegar serves as a crucial processing adjuvant in traditional Chinese medicine, demonstrating the capacity to alter the therapeutic profiles of specific herbal materials. As early as over 2000 years ago during the pre‐Qin period, the ancient medical text *Wushier Bing Fang* documented the use of vinegar as a medicinal excipient in preparing vinegar‐processed Phytolaccae Radix, a traditional Chinese herb with purgative effects (Zhang et al. 2022). In TCM theory, vinegar‐processed herbs leverage the sour flavor of vinegar to enter the Liver Channel, thereby enhancing their therapeutic action on the liver. Vinegar‐processing can enhance properties like promoting blood circulation, activating qi, dissipating blood stasis, and alleviating pain, while also reducing or eliminating toxic components (Jia et al. 2017). This process helps resolve liver blood stasis and dissipate hepatic masses (Zou et al. 2013). Modern research has also demonstrated that vinegar‐processing of TCM enhances their liver‐targeting effects, with the characteristic mechanism primarily manifested by improved hepatoprotective effects, regulation of hepatic microcirculation, modulation of liver enzyme activity, enhanced permeability of active components across hepatocyte membranes, and so on (Peng et al. 2020). Representative examples include: vinegar‐processed Corydalis Rhizoma, which exhibits enhanced blood‐activating and analgesic effects while promoting hepatic and serum accumulation of tetrahydropalmatine (Wu et al. 2014); vinegar‐processed Curcumae Rhizaoma, which protects against concanavalin A‐induced liver injury in mice and exerts anti‐fibrotic effects through TGF‐β1 signaling pathway modulation (Chen et al. 2021). As a food‐grade adjuvant, vinegar demonstrates remarkable efficacy‐enhancing properties in medicinal applications, with its therapeutic benefits firmly grounded in longstanding traditional use. This evidence‐based historical rationale warrants further investigation into its pharmacological mechanisms and clinical potential.

This study confirmed that VCR could significantly improve the therapeutic effects on liver injury by enhancing anti‐inflammatory and antioxidant activities. Notably, existing research indicated that vinegar‐processing of CR offered unique pharmacokinetic advantages. On the one hand, it promoted drug absorption by increasing hepatocyte membrane permeability (Li and Hu 2012), on the other hand, it elevated blood drug concentration by inhibiting the drug efflux effect of P‐glycoprotein (Li and Hu 2013b). In this study, we found that VCR exerts hepatoprotective effects by modulating the TLR4/NF‐κB pathway in the gut‐liver axis. Additionally, research by other scholars has demonstrated that VCR could cooperatively regulate c‐fos protein expression (Li and Hu 2013a) to enhance its efficacy in regulating qi and relieving pain. These findings collectively indicated that VCR exhibited distinctive therapeutic advantages through multi‐target mechanisms. Given the unique value of vinegar‐processing in improving bioavailability, future research should focus on establishing a quality evaluation system for wild CR based on efficacy benchmarks, developing novel processing techniques aimed at enhancing membrane permeability, and exploring integrated industrialization models encompassing “harvesting‐ processing‐ formulation”. Particularly noteworthy is that CR, with its significant pharmacological activity, has long been regarded as a pernicious weed in agricultural production, demonstrating exceptional environmental adaptability and reproductive capacity (Li and Hu 2013a). The findings of this study revealed an important translational medical concept that standardized processing techniques can transform this agricultural waste into medicinal resources with multiple therapeutic values, achieving the concept of “turning waste into treasure”. From a sustainable development perspective, utilizing CR which is a type of “medicinal weed” offers multiple benefits. First, wild resources are abundant and collection costs are low. Second, it does not require additional arable land and thus does not compete with conventional crops. Third, proper harvesting and utilization can help control weed populations and maintain ecological balance. This research strategy, which integrates traditional processing wisdom with modern pharmacological understanding while balancing ecological and economic benefits, not only deepens the comprehension of herbal processing mechanisms but also provides innovative insights into the high‐value utilization of agricultural waste. This holds significant importance for promoting the sustainable development of the TCM industry.

Early‐stage liver disease often manifests as symptoms such as hepatic pain and dyspepsia. Recent studies have found that gut‐liver axis dysfunction plays a critical role in the progression of liver injury. Increased intestinal permeability leads to endotoxin translocation, which directly affects the liver via the portal vein, while intestinal barrier disruption and microbial imbalance exacerbate hepatic inflammation through the TLR4/NF‐κB pathway (Gao et al. 2025). This aligns well with the TCM concept of “simultaneous liver‐spleen regulation”, which emphasizes the close relationship between the liver and digestive system. Our findings demonstrate that VCR mitigated liver injury by restoring intestinal barrier function, rebalancing gut microbiota, and suppressing the TLR4/NF‐κB pathway. VCR exhibited dual regulatory effects on both the gut microenvironment and hepatic inflammation, demonstrating its multi‐target therapeutic advantage which reflected the intrinsic holistic regulation characteristic of TCM. These results provided new insights into liver disease treatment strategies. The doses of CR/VCR extract used in this preclinical study were selected to ensure a clear pharmacological response in the murine model. When converted to human equivalent doses based on body surface area, the effective dose range aligns within a reasonable order of magnitude compared to the established clinical dosage of 6–10 g/day for dried *Cyperus rotundus* L. as per the *Chinese Pharmacopeia*. The apparent difference can be attributed to the use of enriched extracts versus raw herbs, and the paradigm of acute intervention in disease models versus chronic supplementation in clinical practice. Importantly, the identification of α‐cyperone as a key active component provides a direct path for translation. Future development as a nutraceutical would likely utilize standardized extracts with defined α‐cyperone content or purified α‐cyperone itself, which would allow for effective and safe doses in a convenient formulation, thereby enhancing translational feasibility.

The results of this study demonstrated that the content of α‐cyperone in CR. significantly increased after vinegar processing, which is consistent with previous reports (Wang et al. 2025). It is speculated that this phenomenon may be attributed to the thermal effects during processing. On one hand, high temperature may alter the physical state of α‐cyperone within oil chambers or reduce its viscosity, thereby enhancing its extraction efficiency (Chen et al. 1999), on the other hand, the possibility that other constituents were converted into α‐cyperone under heating conditions cannot be ruled out. This hypothesis warrants further verification through isotopic labeling or intermediate identification experiments to track potential compositional transformations. Researched have proved that α‐cyperone, a bioactive sesquiterpene derived from CR, exhibited broad pharmacological properties targeting inflammation, oxidative stress, and neuromodulation. Mechanistic studies demonstrate its ability to suppress pro‐inflammatory mediators including COX‐2 and PGE2 by inhibiting NF‐κB signaling in RAW 264.7 cells (Jung et al. 2013) and BV‐2 cells (Huang et al. 2018), while concurrently activating cytoprotective pathways such as Akt/Nrf2/HO‐1 to mitigate oxidative damage. Additionally, α‐cyperone alleviates paclitaxel‐induced neuropathic pain via norepinephrine pathway modulation (Park et al. 2024) and ameliorates hormone‐linked depression in rat models of mammary gland hyperplasia and chronic stress (Qiao et al. 2023), suggesting its dual role in regulating neuroendocrine‐immune crosstalk. Additionally, our study revealed that α‐cyperone significantly enhances intestinal barrier function, as evidenced by upregulated expression of tight junction proteins ZO‐1 and occludin. It also exerts anti‐inflammatory effects by modulating gut microbiota homeostasis and suppressing the TLR4/NF‐κB signaling pathway. These findings suggest that α‐cyperone holds therapeutic promise for gut‐liver axis‐related disorders. It should be noted that the present gut microbiota analysis establishes associative rather than causal relationships between α‐cyperone‐induced microbial modulation and its hepatoprotective effects. Further investigations employing fecal microbiota transplantation or antibiotic‐treated pseudo‐germ‐free mouse models are warranted to definitively determine whether the observed microbial shifts directly contribute to the hepatoprotective actions of α‐cyperone. Future research should further explore its molecular targets and synergistic mechanisms with other gut‐active compounds to elucidate its pharmacological foundations in protecting both hepatic and intestinal mucosa.

## 5 Conclusion

This study demonstrates that vinegar processing unlocks the hepatoprotective potential of CR by enriching α‐cyperone and enhancing its bioactivity. VCR alleviated liver injury through dual mechanisms including directly suppressing TLR4/NF‐κB‐driven inflammation and oxidative stress in hepatocytes, while concurrently restoring gut microbiota equilibrium and reinforcing intestinal barrier function through upregulation of tight junction proteins. The almost quadrupled α‐cyperone content in VCR underscores vinegar's role as a food‐compatible excipient to amplify bioactive compound extraction, aligning with traditional “vinegar‐processing enhancing liver‐targeted effects” theory. These findings positioned vinegar‐processed CR as a sustainable, food‐grade intervention for liver health, bridging agricultural waste valorization with functional food development. Future studies should explore α‐cyperone's synergistic mechanisms with other gut‐modulating compounds and validate clinical applications in metabolic liver diseases to advance its translation into nutraceuticals or dietary supplements.

## Author Contributions

**Gao Jia‐He:** methodology, data curation, writing – original draft. **Gao Tian‐Hui:** writing – review and editing, funding acquisition, writing – original draft, conceptualization, formal analysis, data curation, project administration. **Liu Jun‐Tong:** supervision, investigation. **Wang Fu‐Chao:** data curation. **Liu Yue‐Han:** writing – original draft, data curation. **Lin Li‐Ting:** methodology, data curation, formal analysis.

## Funding

This study was supported by the National Natural Science Foundation of China (82505032), “Youth Innovation Team Plan” of Universities in Shandong Province (2025KJJ024), 2025 Research Project on Traditional Chinese Medicine Monitoring and Statistics (2025JCTJA16), 2024 University‐level Youth Innovation Team Project of Qilu Medical University (No. X2024QCTD02), and 2025 Shandong Province Youth Research Project for College Students (WL‐SQD25064).

## Ethics Statement

This study was approved by the Institutional Animal Care and Use Committee of Qilu Medical University (permit YXLL2025D065).

## Conflicts of Interest

The authors declare no conflicts of interest.

## Supporting information


**Figure S1:** UPLC‐Q‐Exactive Orbitrap MS analysis of VCR. (A) Total ion chromatographs of VCR, including electrospray ionization positive mode (ESI^+^) and negative mode (ESI^−^). (B) HPLC spectrogram of CR and VCR. (C) Structures of 35 identification compounds.

## Data Availability

The data are available from the corresponding author on reasonable request.
