# Supplementary material for: Unlocking the Hepatoprotective Potential of Cyperus rotundus Through Edible Vinegar Processing: A Study on Functional Ingredient Enhancement
Source: Food Sci Nutr. 2026 May 4;14(5):e71850. doi: 10.1002/fsn3.71850 (PMC13139719; doi:10.1002/fsn3.71850)
Supplement: Supplementary file 1 — Figure S1: UPLC‐Q‐Exactive Orbitrap MS analysis of VCR. (A) Total ion chromatographs of VCR, including electrospray ionization positive mode (ESI+) and negative mode (ESI−). (B) HPLC spectrogram of CR and VCR. (C) Structures of 35 identification compounds. [file FSN3-14-e71850-s001.docx]

****Unlocking the Hepatoprotective Potential of *Cyperus rotundus* through Edible Vinegar Processing: A Study on Functional Ingredient Enhancement****

GAO Jia-He^1,#^，LIN Li-Ting^2,#^，LIU Yue-Han^1^，WANG Fu-Chao^1^，LIU Jun-Tong^1^，GAO Tian-Hui^1,*^

^1^School of Pharmacy, Qilu Medical University, Shandong 255000, China

^2^ School of Pharmacy, Chengdu University of Traditional Chinese Medicine; Key Laboratory of Standardization of Chinese Medicine (Chengdu University of Traditional Chinese Medicine), Ministry of Education; Lab for Innovation & Effective Uses of Chinese Drug Germplasm Resources, Sichuan 611137, China

^#^ These authors contributed equally to this paper.

^*^Corresponding author.

E-mail addresses: gaotianhui@qlmu.edu.cn (TH. Gao)


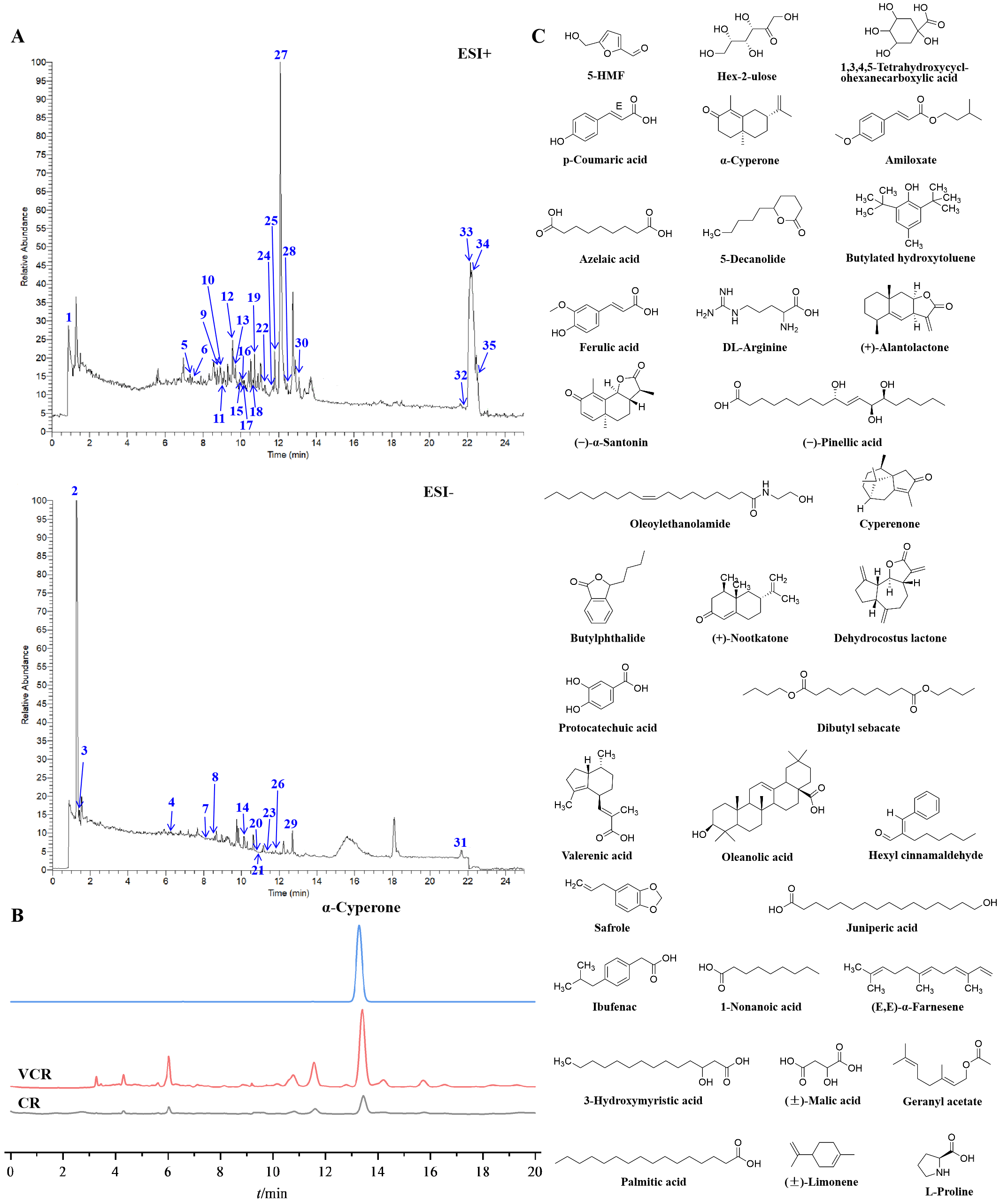


**Fig.S1** UPLC-Q-Exactive Orbitrap MS analysis of VCR. (A) Total ion chromatographs of VCR, including electrospray ionization positive mode (ESI^+^) and negative mode (ESI^-^). (B) HPLC spectrogram of CR and VCR. (C) Structures of 35 identification compounds.
